# Fecal Calprotectin for Small Bowel Crohn’s Disease: Is It a Cutoff Issue?

**DOI:** 10.3390/diagnostics12092226

**Published:** 2022-09-15

**Authors:** Cristina Romero-Mascarell, Gloria Fernández-Esparrach, Cristina Rodríguez-De Miguel, Maria Carme Masamunt, Sonia Rodríguez, Jordi Rimola, Miguel Urpí, Gherzon Simon Casanova, Ingrid Ordás, Elena Ricart, Berta Caballol, Agnès Fernández-Clotet, Julià Panés, Josep Llach, Begoña González-Suárez

**Affiliations:** 1 Endoscopy Unit, Gastroenterology Department, ICMDiM, Hospital Clínic de Barcelona, University of Barcelona, 08036 Barcelona, Spain; 2 Institut d’Investigacions Biomèdiques August Pi i Sunyer (IDIBAPS), Universitat de Barcelona, 08036 Barcelona, Spain; 3 Centro de Investigación Biomédica en Red de Enfermedades Hepáticas y Digestivas (CIBEREHD), 28029 Madrid, Spain; 4 Inflammatory Bowel Disease Unit, Gastroenterology Department, ICMDiM, 08036 Barcelona, Spain; 5 Department of Radiology, Centre de Diagnòstic per la Imatge (CDI), Hospital Clínic Barcelona, University of Barcelona, 08036 Barcelona, Spain

**Keywords:** Crohn’s disease, inflammatory bowel disease, small bowel capsule endoscopy, magnetic resonance enterography, fecal calprotectin

## Abstract

(1) Background: Fecal calprotectin (FC) correlates well with colonic inflammatory activity of Crohn’s disease (CD); data about relation of FC and small bowel (SB) lesions are still contradictory. The main aim was to analyze the relationship between FC levels and SB inflammatory activity in patients with established or suspected Crohn’s disease, assessed by small bowel capsule endoscopy (SBCE) or magnetic resonance enterography (MRE). (2) Methods: Two cohorts of patients were included: 1. Prospective data were collected from patients with established or suspected CD who underwent SBCE and FC (Cohort A); 2. A retrospective cohort of patients who underwent MRE and FC determination (Cohort B). Different cutoffs for FC were tested in both cohorts. (3) Results: 83 patients were included and 66 were finally analyzed. A total of 69.6% had SB lesions seen by SBCE (*n* = 25) or MRE (*n* = 21). FC mean levels were 605.74 + 607.07 μg/g (IQ range: 99.00–878.75), being significantly higher in patients with SB lesions compared to patients without lesions (735.91 + 639.70 μg/g (IQ range: 107.75–1366.25) vs. 306.35 + 395.26 μg/g (IQ range: 78.25–411.0), *p* < 0.005). For cohort A, 25 out of 35 patients had SB lesions and a significant correlation between Lewis Score and FC levels was achieved (R^2^: 0.34; *p* = 0.04). FC sensitivity (S), specificity (E), positive predictive value (PPV), and negative predictive values (NPV) for predicting SB lesions were 80%, 50%, 80%, and 50%, respectively, for FC > 100 µg/g. For cohort B, inflammatory SB activity, measured by MaRIA score, was detected in 21 out of 31 patients (67.7%). Patients with positive findings in MRE had significantly higher values of FC than those with no lesions (944.9 + 672.1 µg/g vs. 221 + 212.2 µg/g, *p* < 0.05). S, E, PPV, and NPV of FC were 89%, 50%, 77.2%, and 71.4% for FC levels > 100 µg/g. The higher sensitivity and specificity of the FC levels for the detection of SB lesions with SBCE and MRE was obtained for an FC cutoff >265 μg/g and >430 μg/g, respectively. (4) Conclusions: FC has a good correlation with the presence of SB lesions, assessed by SBCE and MRE, in patients with established or suspected Crohn’s disease. However, the ideal cutoff is here proven to be higher than previously reported. Multicenter and large prospective studies are needed in order to establish definitive FC cutoff levels.

## 1. Introduction

Crohn’s disease is a chronic inflammatory bowel disease affecting any segment of the gastrointestinal tract, with small bowel (SB) being the most common location (in up to 30% of cases, it is the only segment affected). Proximal SB disease, not accessible with ileocolonoscopy, is present in 10% of patients [1,2]. In these patients, and in the absence of obstructive symptoms or known stenosis, small bowel capsule endoscopy (SBCE) is the first diagnostic tool [3,4,5]. Contrarily, Magnetic Resonance Enterography (MRE) is the initial method of choice in patients with obstructive symptoms or known SB stenosis [6,7,8], being a complementary and/or alternative technique to SBCE although it has less sensitivity for detecting early lesions in the SB [9].

Assessment of inflammatory activity in Crohn’s disease has been traditionally based on signs and symptoms through clinical scores (CDAI, Harvey–Bradshaw), as well as serological biomarkers. However, symptoms are not pathognomonic of Crohn’s disease, and serological biomarkers as C-reactive protein or erythrocyte sedimentation rate have shown a low sensitivity for detecting SB inflammation [10,11,12,13,14]. Therefore, other alternatives as fecal biomarkers have been explored in the last years.

Calprotectin is a calcium-binding protein with antimicrobial, antiproliferative, and proinflammatory properties. Its concentration in the feces is directly proportional to the presence of neutrophils in the intestinal lumen making it useful for detecting inflammation in inflammatory bowel disease in clinical practice [15,16,17].

Fecal calprotectin (FC) has a good correlation with the endoscopic activity in colonic and ileocolonic Crohn’s disease, considering FC levels higher than 250 μg/g as pathological [18,19,20,21,22]. Regarding SB, values higher than 100 μg/g are associated with the detection of lesions by SBCE [23,24,25,26]. However, it is controversial if FC can accurately predict endoscopic activity changes in the SB [27,28,29], and some studies show a worse correlation for ileal CD than for colonic or ileocolonic disease [30,31,32].

Up to now, we have scarce data about the correlation between FC levels and the inflammatory activity in SB measured by MRE [33].

The aim of our study was to assess the correlation of FC levels, clinical scores, and serological markers with inflammatory activity in the SB of patients with established or suspected CD assessed by SBCE or MRE.

## 2. Materials and Methods

### 2.1. Study Design

Two cohorts of patients with established or suspected CD using clinical, radiological, endoscopic, and histological criteria were included: 1. A prospective cohort of patients referred to the endoscopy unit of the Hospital Clinic of Barcelona for SBCE between 2013–2015. The same day of SBCE, the patients were instructed to collect a stool sample within a week, which should be returned to the laboratory for FC analysis (Cohort A). 2. A retrospective cohort of patients selected from a prospectively recorded database who were submitted to a MRE between 2013–2015 and had a FC determination within 3 months (Cohort B).

General exclusion criteria were the following: age less than 18 years old, patients with active colonic CD, perianal disease or pouchitis, treatment with nonsteroidal anti-inflammatory drugs, any contraindication for SBCE, or severe comorbidities. Those patients with FC sample collected more than 3 months apart from the MRE or those who had modified their CD treatment throughout the recruitment period were also excluded. Additionally, in Cohort A, the presence of any contraindication for SBCE was an exclusion criterion.

During the follow-up period, clinical relapse was defined as the presence of gastrointestinal symptoms compatible with CD, requiring hospitalization or a treatment step-up. For the prospective cohort, visits were scheduled every 4–6 months according to daily clinical practice and, for the retrospective cohort, medical records were reviewed.

### 2.2. Small Bowel Capsule Endoscopy Procedure

SBCE was performed with Pillcam SB2 or SB3 (Given Imaging Inc., Yonqneam, Israel), and Endocapsule EC (Olympus, Tokyo, Japan). PillCam SB2 and Endocapsule have a single camera and capture 2 frames per second. PillCam SB3 also has a single camera plus an adaptative frame rate between 2–6 frames per second according to the capsule movement. To improve the visualization of the SB, patients were given 1L of ascorbic acid plus PEG (Moviprep^®^, Norgine VB, Amsterdam, The Netherlands) the day before.

Images were analyzed by 2 gastroenterologists with experience in assessment of SBCE (BG-S and CR-M) with the Rapid Reader 7 software for PillCam and Olympus Endocapsule System 10 for the Olympus capsules, respectively. Standard terminology was used for the description of findings [4]. For the analysis, SB was systematically divided into 3 segments: jejunum, ileum, and terminal ileum.

Severity of SB lesions was graded using the Lewis Score, a quantitative index based on characteristics of villous edema, ulcers, and stenosis. A score lower than 135 was considered as normal or non-clinically significant mucosal inflammatory changes, between 135 and 790 was considered as mild, and ≥790 was defined as moderate to severe disease [34].

Quality of SBCE images was evaluated based on the proportion of the mucosa visualized without debris, liquid, or bubbles. It was categorized as excellent (>90%), good (≥75%), fair (50–75%), or poor (<50%) [35].

Capsule retention was defined as the failure of the passage of the capsule from the GI for more than 2 weeks. Patients with clinical suspected but not confirmed SB stenosis were previously submitted to a dissolvable Agile Patency Capsule (APC).

### 2.3. Magnetic Resonance Enterography Procedure

All examinations were performed using a standardized clinical protocol on a 3.0-T MR unit (TrioTim: Siemens Medical Solution, Erlangen, Germany). Patients fasted for at least 6h and, before MRE, ingested one liter of Polietilenglicol water solution or 2.5% mannitol solution, as intraluminal contrast agent. Protocol of MRE was described elsewhere [9,36,37]. MRE images were assessed by two expert radiologists (SR and JR) with more than 10 years of experience in the evaluation of IBD.

SB wall thickening (>3 mm), edema, hyperenhancement, comb sign (increased mesenteric vascularity adjacent to the inflamed intestinal loop), or presence of ulcers were considered signs of active CD. Strictures were defined as a luminal narrowing less than 10 mm. The presence of fistulas or abscesses was also registered [7,8].

The MaRIA score of each SB segment (jejunum, proximal ileum, and terminal ileum) was calculated to quantify the severity of the SB lesions Active disease was defined as MaRIA ≥7, whereas severe disease was defined as MaRIA ≥11 [38,39].

### 2.4. Biomarkers and Clinical Disease Activity

Clinical disease activity (CDAI) was calculated at the time of SBCE in cohort A whereas the registered index closest to MRE was used in cohort B.

Biomarkers such as platelet count, serum levels of hemoglobin, erythrocyte sedimentation rate (ESR), and C-reactive protein (CRP) were determined by routine laboratory analysis performed at the same period of time.

### 2.5. Fecal Calprotectin

Stool samples were collected at patients’ home in a specific FC collection container, transported to laboratory and stored at −20 °C. FC levels were analyzed by a quantitative immunochromatography test (ScheBo Quick-Prep, Buhlman Laboratories^®^), according to the manufacturer’s instructions [40].

### 2.6. Statistical Analysis

Continuous variables are expressed as mean plus standard deviation and interquartile range. Categorical data are expressed as frequencies and percentages. Chi-square test was used to compare categorical variables. Bivariate correlations were analyzed using Spearman’s correlation coefficient. Performance characteristics of FC levels were calculated using different cutoff. Receiver operator characteristics (ROC) curves were constructed in order to assess the diagnostic accuracy of FC to detect SB lesions. The area under the curve (AUC) and optimal operating point with its sensitivity and specificity and 95% confidence interval s (CIs) were calculated.

A *p* value less than 0.05 was considered statistically significant. All statistical analyses were performed using the Statistical Package for the Social Sciences for Windows software 21.0 (SPSS Inc., Chicago, IL, USA).

## 3. Results

**Figure 1:**
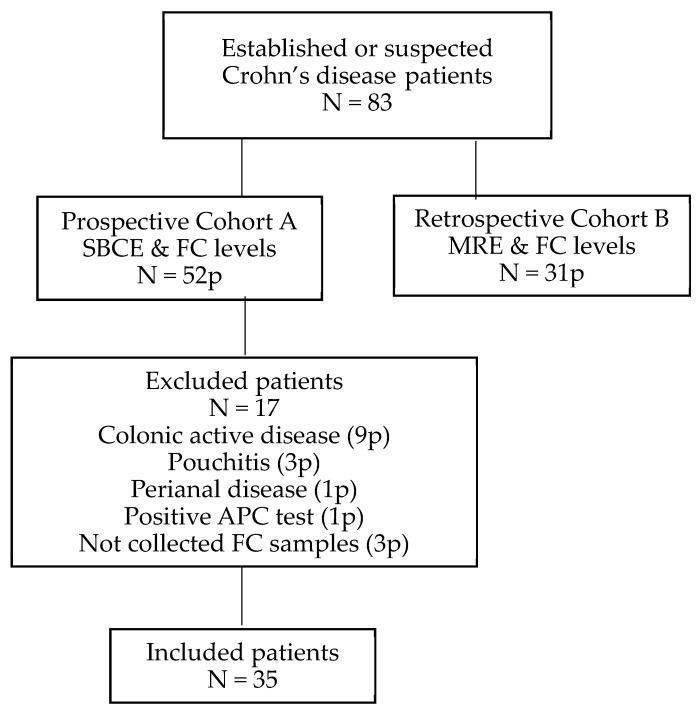
Study flow chart.

A total of 83 patients with established or suspected CD were eligible for the study: 52 patients in Cohort A and 31 patients in Cohort B. In cohort A, 17 patients (32%) were excluded: 3 without FC, 1 with a positive Agile Patency Capsule test, and 13 for other reasons. Finally, 66 patients (35 with SBCE and 31 with MRE) with FC were included. (Figure 1)

A total of 46 out of 66 patients (69.6%) had SB lesions seen by SBCE (*n* = 25) or MRE (*n* = 21). FC mean levels were 605.74 + 607.07 μg/g (IQ range: 99.00–878.75). FC levels were significantly higher in patients with SB lesions compared to patients without lesions (735.91 ± 639.70 μg/g (IQ range: 107.75–1366.25) vs. 306.35 + 395.26 μg/g (IQ range: 78.25–411.0) *p* = 0.007).

### 3.1. Cohort A

Indications for SBCE were suspected CD (*n* = 21) and assessment of SB inflammatory activity in established CD patients (*n* = 14). Demographic, clinical, and analytical characteristics are summarized in Table 1. No cases of capsule retention were registered.

Quality of SBCE images was considered good or excellent in all of patients. Three out of thirty-five patients (8.6%) had an incomplete SBCE study, but in all of them, SB lesions compatible with CD were observed.

SB lesions were detected in 25 out of 35 patients (71.4%): 10 in patients with established CD (10/14, 71.4%) and 15 in patients with suspected CD (15/21, 71.4%). The type of lesions and severity, assessed by Lewis score, are described in Table 2. Sensitivity, specificity, negative predictive value, and positive predictive value related to the different FC cut-offs are shown in Table 3. Using the standard cut-off of 100 μg/g, 20 out the 25 lesions were detected.

FC levels were 503.37 + 541.10 μg/g (IQ range 99–611). Patients with positive findings in SBCE had significantly higher levels of FC than patients without lesions (611 +/− 596.6 µg/g vs. 233 +/− 207.5 µg/g, *p* < 0.05). FC values associated with presence of SB lesions were higher among patients with established CD than in those with suspected disease (700 + 588.63 μg/g (IQ range: 155.25–1390) vs. 584.50 + 625.08 μg/g (IQ range 100.50–809).

A subgroup of 14 patients (41.1%) had proximal lesions (isolate jejunal or jejunal and ileal lesions). No differences were observed regarding FC levels in this group of patients compared with those having isolated ileal lesions (626.9 +/− 563.2 vs. 591.9 +/− 664.2 µg/g; *p* = 0.8).

There were no differences in severity or in serological markers levels between the group of patients with lesions and without (Table 4).

**Figure 2:**
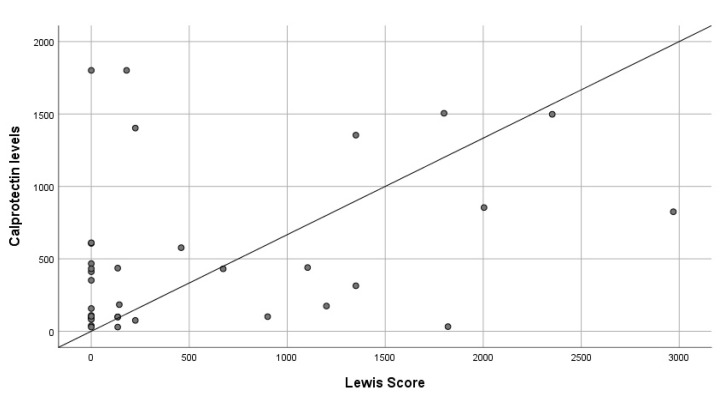
Correlation between fecal calprotectin levels and severity of inflammation measured with Lewis score.

Regarding the severity of lesions, a low positive correlation between Lewis score and FC levels was found (R^2^: 0.34; *p* = 0.04) (Figure 2).

**Figure 3:**
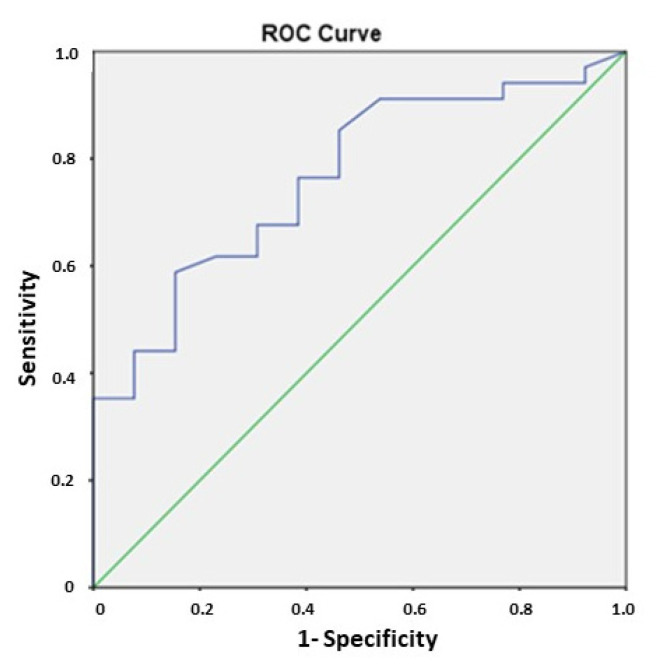
Receiver operating curve analysis for fecal calprotectin levels and presence of small bowel capsule endoscopy lesions.

The area under the curve (AUC) for the diagnostic accuracy of FC to detect SB lesions was 0.76, (95% CI, 0.622–0.908; *p* = 0.005). A cut-off >265 μg/g showed the highest sensitivity and specificity values of FC for the detection of SB lesions with SBCE (sensitivity 67%; specificity 70%) (Figure 3).

### 3.2. Cohort B

A total of 31 patients were included in the MRE cohort. MRE indication was suspected CD in three patients and assessment of SB disease activity in 28 patients with established CD. Demographic, clinical, and analytical characteristics are shown in Table 1.

FC levels were <100 µg/g in 7 patients (41.2%), between 100–500 µg/g in 8 patients, and 16 patients (61.5%) had FC levels >500 µg/g, identifying lesions in MRE in 3 (14.2%), 5 (23.8%), and 13 (61.9%) (*p* = 0.04). Patients with positive findings in MRE had significantly higher values of FC than those with no lesions (944.9 ± 672.1 µg/g vs. 221 ± 212.2 µg/g, *p* < 0.05). Sensitivity, specificity, negative predictive value, and positive predictive value related to the different FC cutoffs are shown in Table 5.

Inflammatory SB activity, measured by MaRIA score, was detected in 21 out of 31 patients (67.7%). Two MRE studies were not evaluated due to lack of SB distension (Table 6).

Otherwise, no differences were observed in CRP and CDAI index between both groups.

**Figure 4:**
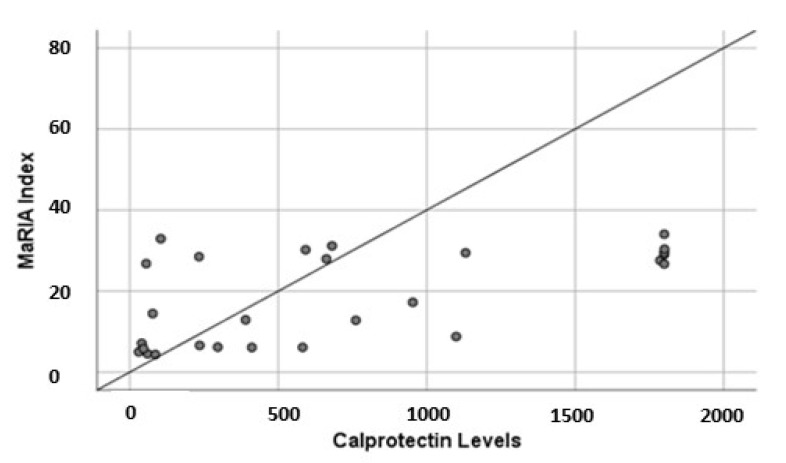
Correlation between fecal calprotectin levels and lesions at magnetic resonance enterography (MaRIA Index).

A moderate positive correlation between MaRIA score and FC levels was found (R^2^: 0.5; *p* = 0.004) (Figure 4).

**Figure 5:**
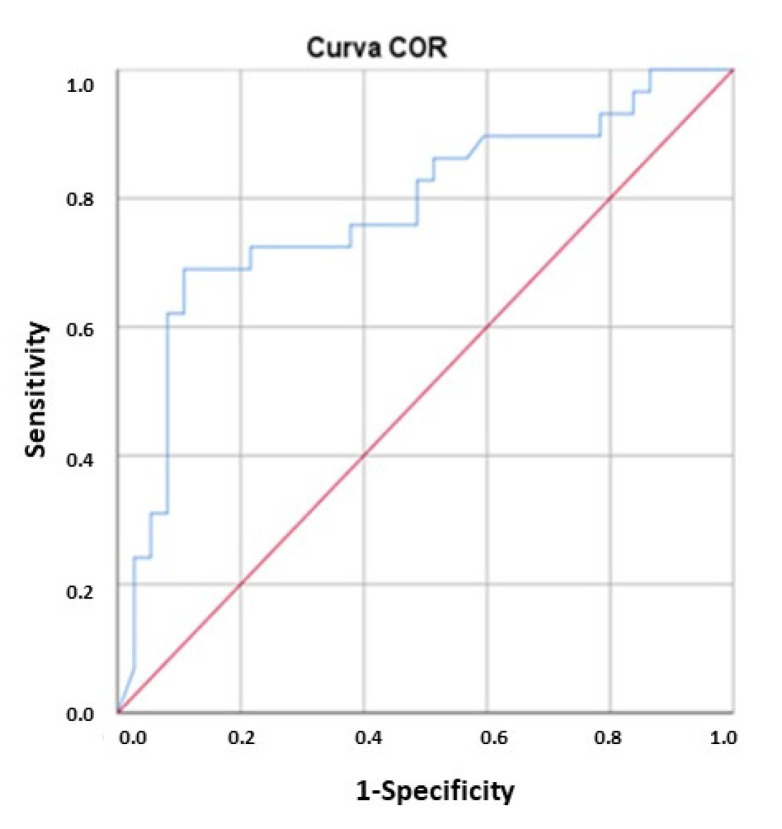
Receiver operating curve analysis for fecal calprotectin levels and magnetic resonance enterography lesions.

Receiver operating curves (ROC) were performed in order to assess the diagnostic accuracy of the FC to detect MRE lesions. The AUC referred to the accuracy in diag-nosing inflammatory activity in MRE was 0.78 (95% CI, 0.663–0.898; *p* = 0.000) show-ing a moderate accuracy for FC to predict lesions in MRE (Figure 5).

The higher sensitivity and specificity of the FC levels for the detection of SB le-sions with MRE was obtained for a FC cut-off > 430 µg/g (Sensitivity 72%, specificity 73%).

### 3.3. Follow-Up

Follow-up was 40.8 + 18.3 months. During this period of time, 16 (24.24%) patients presented a relapse (5 in cohort A and 11 in cohort B). A multivariate analysis was performed and baseline FC levels were not a predictive factor for relapse in the follow-up.

## 4. Discussion

In this study, we analysed the correlation of SB lesions and FC in CD patients assessed by SBCE and MRE. A recent study of our group showed a significantly higher sensitivity of SBCE for detecting superficial and proximal SB lesions compared to MRE (76.6% vs. 44.7%, *p* = 0.001) [9]. However, MRE allows evaluation of transmural lesions and provides an accurate assessment of structuring and penetrating complications, being considered complementary to SBCE in the study of the SB [4,7,8,41]. The present study shows an FC cut-off higher than previously reported, in order to identify lesions in SB for SBCE and MRE (265 μg/g and 430 μg/g, respectively) [18,19,20,21,22]. Two previous meta-analyses and systematic reviews studied the correlation between FC levels and inflammatory activity in the SB so far. Kopylov et al. [24] published a meta-analysis in 2016 that included seven studies (four retrospective); an FC cut-off of 50 μg/g had a high sensitivity (83%), with low specificity (53%) to detect SB lesions assessed by SBCE; a higher specificity was observed with FC > 200 μg/g. The study suggested that patients with FC levels < 50 μg/g, present a low probability to have lesions in the SBCE. This analysis had been after updated by Jung et al. [26] including 14 studies (6 retrospectives) showing similar results; a FC cut-off of 100 μg/g showed a sensitivity and specificity of 73%. They proposed to use this cut-off as a tool to screen SB Crohn’s disease assessed by SBCE. Contrarily, our results showed that a higher cut-off had a better performance for detecting SB lesions by SBCE with a positive correlation with Lewis score. The reason for this discrepancy may be related to the high number of retrospective studies included in the meta-analysis and the use of different diagnostic criteria in the previous publications.

None of the previous published studies differentiate levels of FC depending on suspected or established Crohn’s disease. After performing in our cohort, a separate analysis for suspected and established disease patients, we observed that FC levels associated with presence of SB lesions in the first group of patients were higher than those registered for established Crohn’s disease. Otherwise, we didn’t find any difference regarding FC levels and disease location (isolate jejunal or jejunal and ileal lesions), mentioned by other authors [42].

Few studies have evaluated the correlation between FC and SB lesions assessed by MRE. Recently, Cerrillo et al. [33] performed a prospective study including 120 consecutive patients with Crohn’s disease, and a significant positive correlation was found between MaRIA score and FC levels. A FC cut-off of 166.5 μg/g showed 90% sensitivity and 74% specificity for the diagnosis of inflammatory activity in SB. However, in this study patients with colonic involvement were also included, and higher FC levels could be due to the presence of colonic disease. Contrarily, our study was performed in a population with isolated SB lesions, resulting the higher sensitivity and specificity of the FC for the detection of SB lesions with MRE for a cut-off >430 μg/g.

In the present study, other biomarkers, such as CRP and ESR, have shown to be poor predictors for SB lesions in CD, demonstrating a low sensitivity and specificity, as previously reported in the literature [12,13,14,31].

Previous studies suggested FC as a marker of clinical recurrence. Kennedy A et al. [43] published a retrospective study showing that high FC levels are associated with increased disease progression. In this study, neither SBCE lesions, MRE lesions, baseline Lewis score, or FC level were seen as predictive factors for relapse in the follow-up. This fact may be explained by a small number of patients presenting clinical relapse.

Two main limitations should be pointed out in our study: on one hand, one of the cohorts is retrospective and, secondly, the small sample size may prevent obtaining more definitive results. Strengths of the present study are a partial prospective design and consecutive inclusion of patients with suspected or known small bowel Crohn’s disease.

## 5. Conclusions

This study suggests that FC has a good correlation with the presence of SB lesions, assessed by SBCE and/or MRE, in patients with established or suspected Crohn’s disease. However, the ideal cut-off seems to be higher than previously reported. Multicenter and prospective studies are needed in order to establish definitive FC cutoff levels.

## Figures and Tables

**Table 1:** Demographic, clinical, and analytical characteristics.

	Whole Cohort N = 66	SBCE N = 35	MRE N = 31
Female gender	40 (60.6%)	23 (65.7%)	17 (54.8%)
Age (years)	39.3 +/− 12.7	40.1 +/− 12.2	38.3 +/− 13.4
Active smokers	20 (30.3%)	7 (20%)	13 (41.9%)
Suspected/Established CD	24/42	21/14	3/28
Duration of CD (months)	83.06 ± 106.45	75.86 ± 119.33	91.19 ± 91.02
Previous surgery	10 (15.2%)	5 (14.3%)	5 (16.1%)
Treatment No treatment Anti-TNFs Thiopurines Mesalazine Corticosteroids Anti-TNFs + thiopurines	29 (43.9%) 3 (4.5%) 11 (16.6%) 3 (4.5%) 6 (9.1%) 14 (21.2%)	19 (54.3%) 1 (2.9%) 7 (20%) 0 4 (11.4%) 4 (11.4%)	10 (32.2%) 2 (6.5%) 4 (12.9%) 3 (9.7%) 2 (6.4%) 10 (32.2%)
CDAI score >150 <150	23 (34.8%) 38 (57.6%)	11 (31.4%) 24 (68.6%)	12 (38.7%) 14 (45.2%)
Age at CD onset <18 years 18–40 years >40 years Age of suspected CD	6 (9.1%) 23 (34.8%) 13 (19.7%) 24 (36.4%)	2 (5.7%) 7 (20%) 5 (14.3%) 21 (60%)	4 (12.9%) 16 (51.6%) 8 (25.4%) 3 (9.7%)
Disease location Ileal Colonic Ileo-colonic Upper GI tract Ileal + upper GI tract Suspected CD	29 (43.9%) 1 (1.5%) 7 (10.6%) 1 (1.5%) 4 (6.1%) 24 (36.4%)	9 (25.7%) 1 (2.9%) 1 (2.9%) 1 (2.9%) 2 (5.7%) 21 (60%)	20 (64.5%) 0 6 (19.4%) 0 2 (6.5%) 3 (9.7%)
CD phenotype Inflammatory Stricturing Penetrating Stricturing and penetrating Suspected CD	33 (50%) 3 (4.5%) 3 (4.5%) 3 (4.5%) 24 (36.4%)	11 (31.4%) 1 (2.9%) 1 (2.9%) 1 (2.9%) 21 (60%)	22 (71%) 2 (6.5%) 2 (6.5%) 2 (6.5%) 3 (9.7%)
ESR/hour	12.15 ± 9.61	10.91 ± 6.88	13.63 ± 12.07
CRP (mg/L)	0.39 ± 0.67	0.16 ± 0.34	0.64 ± 0.84
Hb (g/L)	133.03 ± 20.56	135.17 ± 13.33	130.61 ± 26.51

SBCE: small bowel capsule endoscopy, MRE: magnetic resonance enterography. CRP: C-reactive protein; ESR: erythrocyte sedimentation rate, Hb: hemoglobin.

**Table 2:** Small bowel capsule endoscopy findings and Lewis score.

	Patients (*n* = 35)	Established Crohn’s Disease (*n* = 14)	Suspected Crohn’s Disease (*n* = 21)
**Lesions** Isolated jejunum ulcers Jejunum + ileal ulcers Ileal ulcers No lesions	2 12 11 10	1 3 6 4	1 9 5 6
**Severity (Lewis score)** Normal (<135) Mild (135–790) Moderate to severe (>790)	10 15 10	4 4 6	6 11 4

**Table 3:** Performance characteristics of Fecal Calprotectin for the detection of small bowel lesions with small bowel capsule endoscopy.

	S	E	PPV	NPV
FC ≥ 50 µg/g	92%	20%	74%	50%
FC ≥ 100 µg/g	80%	50%	80%	50%
FC ≥ 500 µg/g	40%	90%	40%	90%

S: sensibility; E: specificity, PPV: positive predictive value, NPV: negative predictive value.

**Table 4:** Small bowel capsule endoscopy cohort: clinical, serological biomarkers, and Fecal Calprotectin levels related to small bowel activity.

	Lesions Detected by SBCE (N = 25)	No Lesions by SBCE (N = 10)	*p*
FC mean (µg/g)	611.5 + /− 596.6	233 +/− 207.5	0.009
CDAI score mean (range)	103.2 +/− 102.8	148 +/− 62.9	0.20
CRP (mg/L) mean (range)	0.2 +/− 0.4	0.04 +/− 0.05	0.20
ESR (mm/h) mean (range)	10.1 +/− 6.1	13.57 +/− 9.1	0.25

SBCE: small bowel capsule endoscopy; FC: Fecal Calprotectin; CDAI: Crohn’s Disease Activity Index; CRP: c-reactive protein; ESR: erythrocyte sedimentation rate.

**Table 5:** Performance characteristics of Fecal calprotectin for the detection of small bowel lesions with Magnetic Resonance Enterography.

	S	E	PPV	NPV
FC ≥ 50 µg/g	94.7%	20%	69.2%	66.6%
FC ≥ 100 µg/g	89%	50%	77.2%	71.4%
FC ≥ 500 µg/g	68.4%	80%	86.6%	57.1%

S: sensibility; E: specificity, PPV: positive predictive value, NPV: negative predictive value.

**Table 6:** Magnetic Resonance Enterography findings and MaRIA Score.

	Patients (*n* = 31)	Established Crohn’s Disease (*n* = 28)	Suspected Crohn’s Disease (*n* = 3)
**Lesions** Isolated jejunum Jejunum + ileal Ileal No lesions	0 0 19 12	0 0 19 9	0 0 0 3
**Severity (MaRIA score)** MaRIA ≥ 7 MaRIA ≥ 11 No activity	2 19 8	3 19 4	0 0 3
